# Circuitous embolic hemorrhagic stroke: carotid pseudoaneurysm to fetal posterior cerebral artery conduit: a case report

**DOI:** 10.1186/1752-1947-2-61

**Published:** 2008-02-25

**Authors:** Romy Hoque, Eduardo Gonzalez-Toledo, Alireza Minagar, Roger E Kelley

**Affiliations:** 1 Department of Neurology, Louisiana State University Health Sciences Center, Shreveport, LA 71104, USA; 2 Department of Radiology, Louisiana State University Health Sciences Center, Shreveport, LA 71104, USA

## Abstract

**Introduction:**

The cervical internal carotid artery (ICA) is susceptible to injury through various mechanisms, including dissection, which can lead to pseudoaneurysm formation. Pathological processes affecting the ICA, in association with an ipsilateral fetal posterior cerebral artery (PCA), resulting in parieto-occipital strokes are rarely reported.

**Case Presentation:**

We present a patient with a left PCA territory, presumably embolic, stroke with early hemorrhagic transformation. The identified nidus of the embolus was a carotid artery pseudoaneurysm. Manifestations included right homonymous hemianopsia with right hemiparesis and hemisensory loss.

**Conclusion:**

Our case is unique, and of clinical interest, because it illustrates both the potential anterior-posterior circulation conduit provided by a fetal origin PCA as well as the apparent early hemorrhagic transformation of embolic infarcts that can lead to further confusion from a mechanistic standpoint.

## Introduction

Unexplained hemorrhagic stroke is not infrequently encountered. There are a number of possible mechanisms for such a presentation and it is important to determine the pathogenesis as accurately as possible in an effort to prevent recurrence. Vascular dissection represents tearing of the vessel between the adventia and the intima or the intima and the media. Clinical presentation is usually related to hematoma formation which can compromise the vessel lumen and lead to cerebral infarction as the most common clinical presentation [1]. An alternative mechanism of stroke is aneurysmal outpouching of the affected vessel, resulting in what is termed a pseudoaneurysm, and this can be associated with thrombus formation with the potential for thrombo-embolism into the more distal circulation [2]. Fibromuscular dysplasia (FMD) can contribute to predisposition to vascular dissection [3,4].

Internal carotid artery (ICA) thrombo-embolic disease can be impacted by the presence of a fetal origin ipsilateral posterior cerebral artery [5]. There have been infrequently reported cases of posterior circulation stroke attributed to such a potential conduit [6-9]. We report a patient with a left parieto-occipital hemorrhage in the setting of a left ICA pseudoaneurysm and ipsilateral fetal origin posterior cerebral artery (PCA).

## Case Presentation

A 51 year old right-handed male presented with acute right sided weakness and right homonymous hemianopsia. He reported a recent upper respiratory tract infection with violent cough. Past medical history was pertinent for hypertension and hyperlipidemia treated over the past five to six years with a statin drug and a calcium channel blocking agent. He also recalled a stunning blow to his head, without loss of consciousness, twelve years prior to presentation. His general physical examination was unremarkable. He was afebrile, in normal sinus rhythm and had no ocular or cervical bruit. Neurological examination revealed a dense right homonymous hemianopsia, with a right sided weakness, affecting the face, arm and leg with 4/5 strength, and right hemisensory loss.

**Figure 1:**
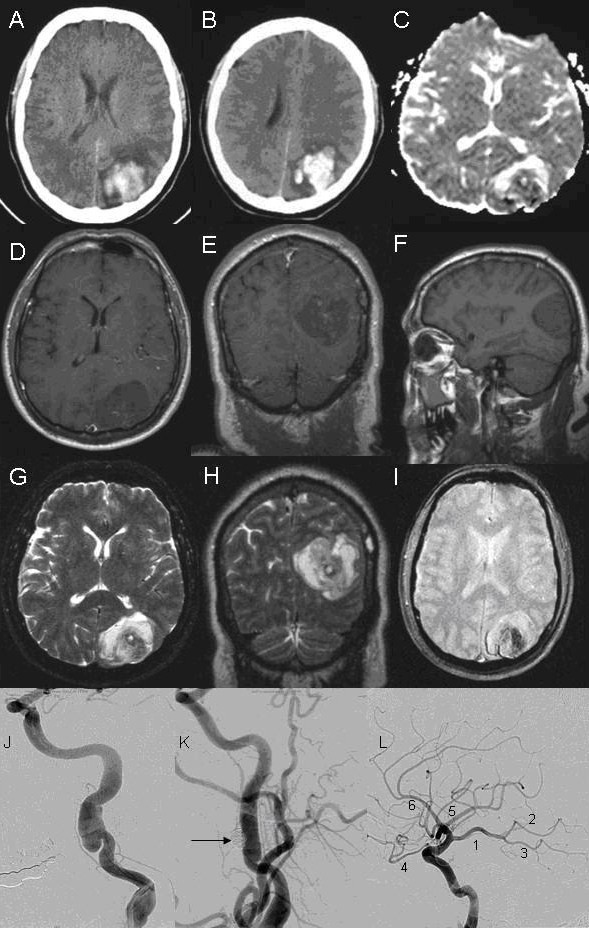
**Neuroimaging of patient with occipital hemorrhage secondary to carotid dissection.** A-B: Computerized tomography (CT) of the head showing left occipital hemorrhage. C: Diffusion weighted imaging (DWI). D, E, F: Axial, coronal, and sagital T1-weighted magnetic resonance imaging (MRI). G, H: Axial and coronal T2-weighted MRI. I: Gradient echo. J, K, L: Angiogram of left carotid artery. J: Left carotid angiogram showing pseudoaneurysm. K: Left carotid angiogram after deployment of coil (arrow) into pseudoaneurysm. L: Left internal carotid artery (ICA) angiogram with branches. 1: Fetal origin posterior cerebral artery (PCA) from ICA. 2–3: Anterior and posterior branches of PCA, respectively. 4: Ophthalmic artery. 5: Middle cerebral artery. 6: Anterior cerebral artery.

Computerized tomography (CT) and magnetic resonance imaging (MRI) of the head one day later revealed an evolving left parieto-occipital primary hemorrhage versus hemorrhagic infarction (Figure 1A–I). Carotid angiogram revealed a left ICA pseudoaneurysm (Figure 1J) in association with a left fetal PCA (Figure 1L). There was some evidence of fibromuscular dysplasia (FMD) and it was felt that his pseudoaneurysm formation was related to this in association with an apparent ICA dissection. He underwent stenting of the left ICA, with coiling of the pseudoaneurysm, one month after presentation (Figure 1K).

He was treated with clopidogrel, 75 mg a day, for six months after his interventional procedure, and has then been maintained on aspirin 81 mg per day for the past two years of observation with essentially full recovery of motor and sensory function on the right side, other than mildly impaired dexterity, but with persistent dense right homonymous hemianopsia.

## Discussion

Roughly 10% to 32% of the population has a fetal origin PCA supplying their parieto-occipital lobes in which the P1 segment is hypoplastic and the PCA is supplied primarily by a larger diameter homolateral posterior communicating artery [5,10]. Other potentially persistent primitive carotid basilar anastomoses include the primitive trigeminal artery, the primitive acoustic (otic) artery, the primitive hypoglossal artery, and the primitive proatlantic artery. The fetal origin PCA anatomic variant provides a potential conduit for emboli from ipsilateral ICA disease [6-9]. To the best of our knowledge, artery-to-artery embolism from cervical ICA pseudoaneurysm to fetal PCA has not been previously reported. Pseudoaneurysm usually develops as a result of trauma, with rupture of the affected artery through the intima and media into the subadventitial plane. The resulting tear is contained by the adventitia forming a pseudoaneurysm. Unlike true aneurysms, pseudoaneurysms do not involve dilatation of the entire vessel wall.

FMD is not only associated with predisposition for initial cervical artery dissection, but also with risk of recurrence [11]. In a study of carotid artery aneurysms [12], 5.5% were attributed to FMD and 6.6% were attributed to trauma. There is not only the potential for ischemic stroke, but also for intracranial hemorrhage as a complication of FMD [13].

Our patient initially presented both with features mimicking an MCA stroke (hemiplegia and hemisensory loss); along with the features typical of PCA stroke (bilateral visual field defects). Our patient's neuroimaging demonstrates an occipital cortical stroke extending to the posterior parietal lobe and sparing the mesencephalon and posterolateral thalamus. The P3 and P4 segment of the PCA (superficial PCA) supply this territory. Previous studies have shown that 17% of patients with pure cortical PCA strokes have face-arm-leg motor deficits, and 23% have sensory deficits in the same distribution [14]. These studies have demonstrated that embolism is the predominant cause of superficial PCA infarction, as presumably occurred in our case.

Early spontaneous hemorrhagic transformation of an underlying embolic ischemic infarction was the suspected pathophysiological mechanism in our patient given the presence of the left ICA pseudoaneurysm as a potential source of emboli. Early hemorrhagic transformation of cerebral infarcts has been linked to embolism, especially from the heart [15]. Artery to artery embolism from ICA pseudoaneurysm to fetal PCA resulting in parieto-occipital ischemia with early hemorrhagic transformation has not been previously reported.

## Conclusion

Our case is unique, and of clinical interest, because it illustrates both the potential anterior-posterior circulation conduit provided by a fetal origin PCA as well as the apparent early hemorrhagic transformation of embolic infarcts that can lead to further confusion from a mechanistic standpoint. Recognition of this anatomic variant can lead to important angiographic diagnostic testing and possibly interventional therapy with newer endovascular stenting techniques.

## Competing interests

The author(s) declare that they have no competing interests.

## Authors' contributions

All authors contributed to each stage of this work. This means that RH, EGT, AM, and REK all have: (1) made substantial contributions to conception and design, or acquisition of data, or analysis and interpretation of data; (2) been involved in drafting the manuscript or revising it critically for important intellectual content; and (3) given final approval of the version to be published.
